# Association between Functional Movement Screen Scores and Athletic Performance in Adolescents: A Systematic Review

**DOI:** 10.3390/sports10030028

**Published:** 2022-02-22

**Authors:** Katie Fitton Davies, Ryan S. Sacko, Mark A. Lyons, Michael J. Duncan

**Affiliations:** 1 Centre for Sport, Exercise and Life Sciences, Coventry University, Coventry CV1 5FB, UK; k.fittondavies@ljmu.ac.uk; 2 Research Institute for Sport and Exercise Sciences, Liverpool John Moores University, Liverpool L3 5UG, UK; 3 Department of Health and Human Performance, The Citadel, Charleston, SC 29409, USA; rsacko@citadel.edu; 4 Sport and Human Performance Research Centre, University of Limerick, V94 T9PX Limerick, Ireland; mark.lyons@ul.ie

**Keywords:** movement, motor skill, children, youth, sport performance, testing, athletic development, screening

## Abstract

This study systematically reviews the literature examining the relationship between Fundamental Movement Screen (FMS©) scores and athletic performance in youth. We searched English-language papers on PubMed/MEDLINE, SportsDiscus, CINAHL, and EBSCO for the following inclusion criteria: Participants aged between 11 and 17 years, studies had to include the Functional Movement Screen© (FMS©) and at least one of the following performance outcomes, highlighted by athletic development models (i.e., long-term athletic development (LTAD), youth physical development (YPD)): agility, speed, power, strength, endurance, and balance (YPD), fitness (LTAD), or sport-specific skill (LTAD and YPD). A total of 3146 titles were identified, with 13 relevant studies satisfying the inclusion criteria after full-text screening. The results of this systematic review suggest that children and youth who score highly on the FMS© also tend to have better scores for agility, running speed, strength, and cardiovascular endurance. The strength of associations was weak to moderate in nature. Only one study was considered or controlled for biological maturation in their analysis. These results provide evidence that, while there is a relationship between FMS© scores and tests of athletic performance in youth, they are not the same thing and should be considered conceptually different constructs.

## 1. Introduction

Over the last decade, there has been an increased interest in the importance of functional movement for health and sports performance [1]. Functional movement skills (FMS) have been proposed as bodily movements characterised by adequate joint and muscle function, and by a movement efficiency that minimises the risk of injury [2,3]. It should be noted early on that in the movement literature, “FMS” has been used for ‘functional movement skills’ [2,3] and ‘fundamental movement skills’ [4]. The two types of movement are different, but they also overlap [5]. Fundamental movement skills are considered the building blocks of more advanced movement, such as those found in sport [6,7], consisting of locomotor (e.g., run, skip, jump), object control (e.g., catching, kicking, striking), and stability skills (e.g., dynamic and static balancing) [8]. Functional movement skills, however, refer to the movement pattern that underpins all other movements [5] and is needed for successful engagement in health-enhancing physical activity (PA) and sports performance [9]. The concept of functional movement is well established in the literature [10,11]. Consequently, the assessment of functional movement, and gaining an understanding of the extent that functional movement relates to other health and sports performance outcomes and injury risk [10,12,13], have become key areas of interest for sports medicine and sports science practitioners.

The assessment of functional movement typically involves the measurement of postural control, stability, flexibility, neuromuscular coordination, and balance [2,3,14]. While there are a variety of tools purported to assess functional movement, including the Landing Error Scoring system [15] and marker-based biomechanical assessments [10], the Functional Movement Screen© (FMS©) has been established as the predominant tool used for functional movement assessment [16]. The FMS© is comprised of seven movements aimed at the assessment of mobility, flexion, extension, and stability [2,3]. The squat, lunge, and hurdle step tests are described as higher-level patterns which are proposed to examine the three essential foot positions taken up in sports. The rotary stability and press up tests are known as transitionary patterns and predominantly assess tri-planar and sagittal stability. Finally, the lower-level mobility patterns of the body are assessed by the active straight leg raise and the shoulder mobility tests [17]. In the current study, we use the term FMS to refer to functional movement skills and the term FMS© to refer to the functional movement screen.

Each item in the FMS© is scored on an ordinal scale from zero to three, with three representing optimal movement and zero is scored when a test is discontinued due to pain. Scores for each item are summed across all 7 movements with a maximum possible score of 21, reflecting better functional movement. Further, also included in three of the FMS© movements (shoulder stability, lumbar flexion, and trunk extension) is a movement dysfunction clearing test to identify pain. If pain is present, the participant receives a zero even if a score of three was initially obtained. Latterly, a modification of the FMS©, incorporating a 100-point scoring scale, has been developed [18] on the basis that it provides a more sensitive tool by itemising each movement and scoring bilateral movements separately. The use of the 100-point scale, however, has not been utilised to date in studies examining the association between FMS© scores and athletic performance (e.g., muscular strength, aerobic fitness, agility). The FMS©, employing the 0–21-point scale, has, however, been widely used with a variety of populations, such as athletic groups [19] youth athletes [20], older adults [21] and children [5]. Monitoring functional movement competencies using the FMS© is useful, as functional movement competency provides the foundation to lifelong PA and sport participation [22]. Movement competency is scaffolded by stability and neuromuscular control (elements captured in the FMS©), which are essential for all forms of movement, sport, and exercise [7,23,24].

Understanding how functional movement might impact PA and sports performance in youth is important in developing positive trajectories of movement aligned with lifelong PA and sports participation. Recently, a systematic review by O’Brien et al. [25] provided an important overview of the FMS© in children and adolescents. This work was undertaken in the context of PA specialists working in physical education and highlighted differences in FMS© scores between boys and girls and between primary and secondary school-aged children. In particular, there was over four times greater variability in FMS© scores for studies with secondary school-age children, possibly signalling potential developmental and maturational effects during the adolescent period [25]. O’Brien et al. [25] also highlighted a consistent and negative association between FMS© scores and BMI, and greater variability in FMS© scores during adolescence, potentially due to maturation effects that were not assessed in their review. Understanding normative values for FMS© in children and adolescents is a useful step forward, but there remain key gaps in understanding. These gaps relate to functional movement and how it might associate with a range of outcome measures (e.g., strength, speed, agility) aligned with PA, and sports performance. Unlike the recently published review by O’Brien et al. [25] which focused on age and sex-related differences in FMS© scores as a means to provide normative data, the present study systematically reviews the literature which examines the association between FMS© scores and athletic performance in youth.

Based on Cook’s model [9] of movement development, where functional movement underpins functional performance, prior studies have examined short-term associations between FMS© scores and multiple aspects of fitness and sports performance including speed [24,26,27], agility [20,27,28], balance [26], and strength performance [20,28] in youth. The development of functional movement during childhood and youth is also a key feature within long-term athletic development (LTAD) models for youth sports performance. Briefly, the LTAD model offers a strategic approach to athletic development as it considers the maturational status of the child [29]. “Windows of opportunity” exist within the LTAD model, where children and adolescents are more sensitive to training-induced adaptation. Moreover, according to the LTAD model, a failure to use these windows will result in limited future athletic development. The Youth Physical Development (YPD) model [30] extends the LTAD by offering an individualised (one for girls, one for boys) overview of physical development, identifying when to train certain athletic performance elements (e.g., strength, speed, agility), and why it should be emphasised at this age, effectively de-emphasising the importance of the LTAD’s “windows of opportunity”. Understanding the relationship between functional movement and athletic performance could add greatly to our understanding of LTAD models, such as that proposed by Lloyd and Oliver [30], which consider fundamental motor skill development with LTAD but not FMS. 

Important athletic performance tasks, identified by the LTAD and YPD models, include agility, speed, power, strength, and endurance (note: although mobility is also included in the YPD, it is never viewed as a developmental focal point, and as a consequence may not be considered as important as the other elements). Agility is defined as a rapid whole-body movement with a change of velocity or direction in response to a stimulus [31], although a majority of literature still consider agility as a change of direction and speed [31], which can be measured by change of direction assessments, such as the agility *t*-test [32]. Speed can be considered as sprinting ability and can be measured through timed 5 m, 10 m or 30 m sprint tests, or kicking speed, which can be measured through the use of a radar gun (e.g., Stalker ATS II Radar System; Applied Concepts, Inc., Plano, TX, USA). Power (the ability to perform fast, forceful, and propulsive movements) including muscular power, can be indirectly assessed through the vertical jump to measure lower-limb muscular power [33]. Strength (the ability to exert force to overcome resistance) includes eccentric and concentric strength, measured through a squat jump with mobile contact map, as well as grip strength, measured by a hand dynamometer. Cardiovascular endurance (the ability of the heart, lungs, and blood vessels to deliver oxygen to body tissue) can be assessed through the FitnessGram^®^ 20 m PACER test, or as muscular endurance (the ability of muscle or muscle groups to repetitively contract against force over an extended period of time), which can be assessed through the FitnessGram^®^ curl-up test. 

To date, there has been no attempt to synthesise evidence relating to FMS© scores and athletic performance tasks. Understanding how FMS© scores are associated with athletic performance tasks is key to developing evidence-based training programmes, talent identification, and development programmes in youth sport. Robust synthesis of FMS© scores and correlates related to athletic performance specific to youth is needed to provide evidence-based strategies for athletic development in the early stages of development. This is because LTAD models to date take into consideration the maturational status of the child in an effort to devise and put forward more strategic approaches to athletic development and performance [29,30]. Most LTAD models allude to critical “windows of opportunity” as youth develop, where children and adolescents are more sensitive to training-induced adaptation. Therefore, it is critical that the relationship between FMS© and athletic performance in youth is better understood so as to fully consider the importance of FMS© in terms of future LTAD models. The current study aimed to answer the question of how, and to what extent FMS© scores are associated with athletic performance tests in youth. 

## 2. Materials and Methods

The systematic search in this study was conducted in accordance with the preferred reporting items for systematic reviews and meta-analyses (PRISMA) guidelines [34].

### 2.1. Eligibility Criteria

Studies were included if the participants were aged between 11 and 17 years. Eleven years of age was selected as the lower boundary as at this age, a rapid pubertal change begins, signifying early adolescence [35]. Seventeen years of age was selected as the higher boundary as it limits the studies to high school, as collegiate samples include non-adolescent populations. Participants also had to be fit and healthy at the onset of the study to be included (i.e., no rehabilitation studies), as a previous injury may significantly affect FMS composite scores [36]. Studies had to include the Functional Movement Screen© (FMS©) and at least one of the following performance outcomes, highlighted by athletic development models (i.e., LTAD, YPD): agility, speed, power, strength, endurance, and balance (YPD), fitness (LTAD), or a sport-specific skill (LTAD and YPD). 

Studies were excluded if a screen of functional movement that was not the FMS© was used or if there was no investigation into the relationship between functional movement and the performance outcomes listed above. Studies that used a functional training method were excluded unless they investigated the relationship between functional movement and performance outcomes prior to a functional training period. This exclusion criterion relates to the scope of this study, specifically, the investigation of the relationship between functional movement and performance outcomes and not the effectiveness of an intervention. Studies published in languages other than English were only excluded if a translation was unattainable. Studies were excluded if either of the outcomes of interest (i.e., functional movement and performance outcomes) were not measured (i.e., only one or neither) nor not reported (i.e., measured but the investigation did not include the relationship between the two outcomes of interest).

### 2.2. Information Sources

**Figure 1:**
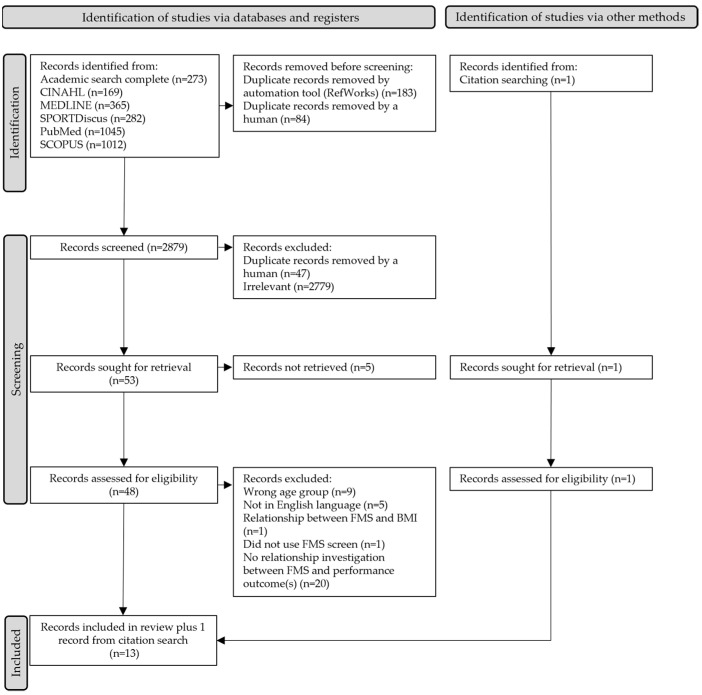
Studies included in this systematic review.

On the 31 March 2021, a comprehensive literature search was conducted using the Elton B. Stevens Company (EBSCO) search engine, which includes the following databases: Academic Search Complete, Cumulated Index to Nursing and Allied Health Literature (CINAHL), Medline and SportsDiscus, as well as PubMed on the 28 April 2021 (Figure 1). The systematic search examined papers from the 1 January 2006, as this was when the Functional Movement Screen© was created, to the 31 March 2021 and 28 April 2021, respectively.

### 2.3. Search Strategy

The key search terms used in each database were as follows: (Functional movement screen* OR “functional movement screen” OR FMS) AND (Teenager* OR child* OR youth* OR adolescent* OR young adult*) AND (Power OR fitness OR jump* OR speed OR strength OR balance OR agility OR sport* OR “physical performance” OR “athletic performance” OR “muscular endurance” OR “cardiovascular endurance”). The search strategy was peer-reviewed by an experienced information specialist affiliated with the team. All studies were exported from their respective databases to RefWorks. Duplicates identified by RefWorks were removed and any remaining duplicates were individually removed. 

### 2.4. Selection Process

Two researchers (KFD, MD) independently reviewed 3146 titles and abstracts. In case of disagreement, consensus on the studies to be screened for full-text was reached by agreement. Next, two researchers (KFD, MD) independently screened full-text studies for inclusion. Again, in the case of any conflicts, consensus on whether to include or exclude was reached through discussion. 

### 2.5. Data Collection Process

Authors of studies that were initially unretrievable were contacted by a researcher (MD) through email to obtain copies of those studies. Once all studies were obtained, data were collected by one researcher independently (KFD) and inputted into an Excel sheet, which was then verified by another researcher (MD).

### 2.6. Data Items

Eligible outcomes were broadly categorised as follows in Table 1:

Any measure of athletic performance was eligible for inclusion. Results could be presented as a total score that provides a composite measure across multiple areas of functional movement (e.g., FMS© composite score), sub-scales that present a measure of domain-specific functional movement (e.g., FMSmove, FMSstab, FMSflex), or individual movements (e.g., deep squat, hurdle jump, etc.) Results from cross-sectional studies were eligible. Longitudinal studies and intervention studies were also eligible only if results were provided from time points that would allow for cross-sectional interpretation (i.e., not a change score, no intervention) or presented baseline measurement from before an intervention (i.e., baseline). 

It was anticipated that individual studies might report data for multiple performance outcomes. For example, speed is often measured via a 10 m, 20 m and 30 m sprint trial(s). It was also anticipated that studies may use different measures of the same performance outcome, for example, agility with the maximal effort 20 m shuttle run or the agility *t*-test. Correlational relationships were extracted between FMS© and performance outcomes. 

The collected data included:

- The report: author, year;
- The study: sample size, country, study design;
- The participants: sex, age, performance status, sport;
- The results: statistical analysis, confidence intervals, effect sizes, FMS© inter-rater reliability, control for maturation.

### 2.7. Study Risk of Bias Assessment

Two researchers (KFD, MD) independently assessed the risk of bias in the included studies. It was decided to use the AXIS tool [37], which is a popular critical appraisal tool specifically designed to assess the quality and risk of bias in cross-sectional studies. As the inclusion criteria dictated that all studies need at least a baseline cross-sectional measurement of FMS© scores and athletic performance outcomes, with the exclusion of longitudinal studies focusing on change over time, this tool was deemed appropriate for the purpose of this systematic review. 

The AXIS tool was developed through three rounds of Delphi poll, which included participants from epidemiology and public health. The resultant tool consists of 20 items that span the introduction, methods, results, discussion sections of studies as well as an ‘Other’ category, which explores reporting of any conflict of interest and ethical procedures. The AXIS tool does not include a numerical scale that can produce a score. Alternatively, the tool aims to assess each characteristic of a study in an accumulative manner. Researchers answer each question with either ‘yes’, ‘no’, or ‘don’t know.’ The two researchers in this study met to discuss any conflicts in coding until a consensus was achieved. 

### 2.8. Effect Measures 

It was anticipated, due to the cross-sectional nature of studies that were included in this systematic review, that a type of correlational analysis would be conducted between FMS© scores (total, sub-constructs, and/or individual movement scores) and athletic outcomes (e.g., speed, agility) in a number of the included studies. As such, the strength of associations would be taken into account to assess the associations between outcomes across studies. Synthesis of these studies would include an interpretation of results according to the strength of associations (e.g., weak, moderate, strong) cited in each individual study. 

### 2.9. Synthesis Methods

Three considerations within this systematic review have the potential for variety; (1) performance outcomes can be measured through different means, (2) the wide number of performance outcomes included within this systematic review, and (3) the acceptance that studies would include more than one performance outcome. As such, we attempted to categorise the included studies into general athletic outcomes determined by the LTAD and YPD models: power, speed, agility, strength, balance/stability, endurance, flexibility, and sport-specific skills. A summary approach to the synthesis of results was undertaken for this systematic review.

## 3. Results

### 3.1. Study Selection

The search strategy identified 3146 potentially relevant studies; 267 were removed as duplicates. Of the 2879 remaining studies, a further 47 studies were identified as duplicates and removed, and 2779 studies were judged to be irrelevant, leaving 53 studies to be reviewed. The authors of five studies were contacted in order to include their studies in the review; however, these five studies could not be retrieved. Therefore, 48 studies were screened at full-text. Of these 48 studies, 36 did not meet the inclusion criteria and were removed. One article was identified through citation searching, which met the inclusion criteria, resulting in 13 studies deemed suitable for this systematic review (Figure 1). 

The primary reasons for the exclusion of the 36 studies at full-text review were because (1) relevant age-based outcomes (*n =* 9) were not discernible, (2) studies did not directly investigate the relationship between FMS© scores and performance outcomes (*n =* 20), (3) studies were not in English (*n =* 4), (4) studies included injured participants (*n =* 1), (5) studies did not use the FMS© (*n =* 1), or (6) studies investigated FMS© and Body Mass Index only (*n =* 1). For example, Molina-Garcia et al. [38] investigated the association between functional movement and fitness. However, although they included 11–12-year-olds, which was appropriate for this systematic review (ages 11–17), they presented their association results including their full age range (8–12 years). This presentation of results meant that we could not extract the relevant associations for this systematic review. In another example, Klusemann et al. [39] investigated adolescents with a mean age of 14 ± 1 and 15 ± 1 (males and females, respectively), used the FMS©, and included performance outcomes. However, they did not present results investigating the association between FMS© scores and performance outcomes and were therefore excluded. 

### 3.2. Study Characteristics

Characteristics of the 13 included studies can be found in Table 2. In terms of the 13 studies identified (Figure 1), all were published between 2015 and 2021. Geographically, studies were conducted in Australia [40], Italy [41], Portugal [42], Slovakia [43], South Africa [44], Taiwan [45], Turkey [46,47], United Kingdom [20,48] and the United States of America [27,49,50]. The mean age range across these studies was 11 to 17.40 years, with 2 studies [46,48] exclusively reporting age for boys and girls separately.

Five studies only included male participants [20,40,41,43,50], five studies included a mix of males and females [27,46,47,48,49], while three studies did not explicitly describe the gender of their participants [42,44,45]. As such, overall, there were 1649 participants across the studies (minimum = 20, maximum = 981), with 185 reported to be female (11.22%). Studies included participants from a variety of sports: American football (*n =* 2), Australian football (*n =* 1), baseball (*n =* 1), basketball (*n =* 1), handball (*n =* 1), karate (*n =* 2), lacrosse (*n =* 1), soccer (*n =* 6), swimming (*n =* 2), volleyball (*n =* 2), and one categorised as ‘other’. Most studies (*n =* 11) were cross-sectional in nature; however, seven studies did not explicitly state this, and one study included a longitudinal design (although this aspect of the study was not included in the data extraction or synthesis). Two studies were reported to be “observational”.

All studies reported a total FMS© score (Appendix A); however, one study presented this total score according to status (elite v sub-elite; [41]), three studies presented the total score according to age [20,42,43], three studies presented the total score according to gender [27,46,49], with one study presenting by both gender and overall sample [48], while five studies presented the overall total score for the sample [40,44,45,47,50]. One study investigated the sub-constructs of the FMS© (FMS_move_, FMS_stab_, FMS_flex_; [41]), five studies only investigated the total FMS© score [20,27,47,48,50], and seven studies investigated the individual movements of the FMS© [40,42,43,44,45,46,49]. The most commonly investigated athletic performance category was power (*n =* 9), followed by speed (*n =* 7), agility (*n =* 6), strength (*n =* 4), balance (*n =* 4), endurance (*n =* 3), flexibility (*n =* 2), and sport-specific skills (*n =* 1). 

### 3.3. Risk of Bias in Studies

The assessment of the study of the risk of bias is presented in Table 3. The range of “yes” answers to the tool’s questions ranged from 5 [47] to 15 [49], with all other studies accumulating 10 or above. The items where all studies were categorised as “yes” included the provision of clear aims, appropriate design, appropriate assessments to determine the aim of the study, appropriate assessments for the participants, and ethical consent. The range of “no” answers to the tool’s questions ranged from three [40,49] to nine [47], with all other studies accumulating seven or below. The items where all studies were categorised as “no” included: a lack of sample size justification, no measures were undertaken to address and categorise non-responders, and no description of non-responders. The range of “don’t know” answers to the tool’s questions ranged from one [48] to six [47], with all other studies accumulating three or below. The only item where all studies were categorised as “don’t know” was not knowing about the relationship between response rate and non-responder bias. 

### 3.4. Results of Individual Studies

When undifferentiated by sex, FMS© scores ranged from a mean of 12.18 ± 2.02 [45] to a median of 16.00 [50]. Across the five papers that did differentiate by sex, females consistently scored higher than males. Female scores ranged from 14.10 ± 1.88 [49] to 17.71 ± 1.65 [46] and male scores ranged from 12.62 ± 3.06 [49] to 16.13 ± 2.32 [46]. Three papers differentiated FMS^©^ scores by age and consistently demonstrated that older ages had higher FMS scores. One paper differentiated their sample by elite status [41] and found the elite group to score higher than the sub-elite group. However, the article by Campa et al. [41] included athletes who were around 16 years of age and demonstrated lower FMS© scores in comparison to the comparative age groups in studies by Silva et al. [42], Lloyd et al. [20] and Bakal’ár et al. [43]. 

### 3.5. Results of Syntheses

As previously explained, the athletic outcomes for each included study were categorised into power, speed, agility, strength, balance, endurance, flexibility, and sport-specific skills. As such, the results from each study will be presented within this structure, starting with the total FMS© score and then individual FMS© movements, starting with power. All correlations can be found in Appendix B (total FMS© and performance outcomes) and Appendix C (individual FMS© and performance outcomes). Strength of association across studies using Pearson’s correlation or Spearman’s rho was determined using the following categories: 0–0.3 (weak); 0.3–0.7 (moderate); 0.7–0.9 (strong); 1 (perfect) following recommended guidelines [51]. 

Five studies were omitted from the table that describes the relationships between total FMS© scores and performance outcomes (See Appendix B). Reasons for this include: three studies did not test the relationship between total FMS© score and performance outcomes, only the individual movements [44,45,49], one study reported no significant correlations between total FMS© and performance outcomes and subsequently did not report those particular non-significant results [43], and one study also found non-significant results; however, they presented negative little or no correlation between these variables (*r* = −0.001 to −0.146; [42]).

#### 3.5.1. Power

Of the 11 power-based outcomes, only 1 power-based performance outcome, the squat jump was associated with the total FMS© score (Appendix B). This positive association was found across two studies [20,47]. Both studies had a combined sample of 62 participants (30 and 32). Lloyd et al. [20] was categorised as one of the least biased studies while Yildiz [47] was categorised as the highest biased study. Both studies reported a moderate strength of association (*r* = 0.52−0.66). 

Bennett et al. [40] found weak positive associations between the trunk stability push-up (TSPU) and countermovement jump (*r* = 0.22, *p* < 0.05), the unilateral running jump height left (*r* = 0.18, *p* < 0.05), and right (*r* = 0.18, *p* < 0.05). Bennett et al. [40] was one of the least biased studies and included 981 participants. Lloyd et al. [20] reported a moderate positive association between squat jump and rotary stability (*r* = 0.65, *p* < 0.01).

#### 3.5.2. Speed

Across the 13 studies, there were 10 speed-based outcomes. There were a number of associations between total FMS^©^ and speed-based outcomes across four studies. Campa et al. [41] found moderate negative associations between repeated sprint ability best time (*r* = −0.58, *p* < 0.001) for total FMS^©^, and for FMS_move_ (repeated sprint ability mean time (RSAM), *r* = −0.36, *p* < 0.06; repeated sprint ability best time (RSAB), *r* = −0.55, *p* < 0.001). Campa et al. [41] had 36 participants and was judged to be relatively low in bias. Two studies used swim time as a speed-based performance outcome where Erkan et al. [46] found no association for males or females and their 200 m swim time and total FMS© scores. In contrast, Bond et al. [48] found a significant main effect for swim speed (fast v slow; F = 8.20, *p* = 0.005), indicating that faster swimmers had better FMS© scores. They also found a moderate negative association between FMS© and 100 m freestyle swim (*r* = −0.333, *p* < 0.05). Erkan et al. [46] and Bond et al. [48] had 93 and 50 participants, respectively; Bond et al. [48] were relatively lower in bias in comparison to Erkan et al. [46]. Bennett et al. [40] reported a weak negative association between total FMS^©^ and 5 m sprint time (*r* = −0.13, *p* < 0.05).

Looking at the individual movements of the FMS©, there was a moderate negative association between the 5 m sprint and the deep squat for 15-year-olds (*r* = −0.064, *p* = 0.02; [43]). Bennett et al. [40] found a weak negative association between the hurdle step and 20 m sprint time (*r* = −0.14, *p* < 0.05). There was also a weak but significant negative association between the in-line lunge and 5 m and 20 m sprint times (*r* = −0.116, *p* < 0.05, *r* = −0.0134, *p* < 0.05, respectively; [40]). Bakal’ár et al. [43] found a moderate negative association between the 5 m sprint and TSPU for 11-year-olds (*r* = −0.50, *p* = 0.04) and a weak negative association between the 5 m (*r* = −0.12, *p* < 0.05) and 20 m sprint times (*r* = −0.16, *p* < 0.05).

#### 3.5.3. Agility

Three agility-based outcomes were sourced from the studies. Three studies found negative associations between their respective agility-based performance outcomes and total FMS© scores. Lloyd et al. [20] and Kramer et al. [27] found moderate negative associations with reactive agility (*r* = −0.54, *p* < 0.01; [20]) and agility (*r* = −0.47, *p* < 0.01; [27]). Bennett et al. [40] found a weak negative association with agility (*r* = −0.13, *p* < 0.05). 

With respect to the individual movements of the FMS©, Lloyd et al. [20] found a moderate negative association between reactive agility and the deep squat (*r* = −0.40, *p* < 0.05). Bennett et al. [40] found a weak negative association between agility and the hurdle step (*r* = −0.16, *p* < 0.05). Lloyd et al. [20] found a moderate negative association between reactive agility and the in-line lunge (*r* = −0.60, *p* < 0.05) and they also found that the in-line lunge predicted reactive agility (adjusted R^2^ = 0.38), with maturation explaining further variation (adjusted R^2^ = 0.46). Conversely, Bennett et al. [40] found a weak negative association between agility and the in-line lunge (*r* = −0.12, *p* < 0.05). Only Lloyd et al. [20] found a moderate negative association between reactive agility and the active straight leg raise (*r* = −0.59, *p* < 0.01). A few negative associations were found between agility-based performance outcomes and the trunk stability push-up. Bakal’ár et al. [43] found a moderate negative association between agility on the preferred side and TSPU (*r* = −0.61, *p* = 0.01). Chang et al. [45] found a moderate negative association between agility and TSPU (*r* = −0.57, *p* < 0.05). Lloyd et al. [20] found a moderate negative association between reactive agility and rotary stability (*r* = −0.58, *p* < 0.01) while Bennett et al. [40] again found a weak negative association between agility and rotary stability (*r* = −0.12, *p* < 0.05).

#### 3.5.4. Strength

There were six strength-based outcomes across the studies. Two studies investigated a strength-based outcome and association with total FMS© scores. Lloyd et al. [20] found a large positive association between reactive strength specifically, and total FMS© (*r* = 0.74, *p* < 0.01). Yildiz [47] found a moderate positive association with back and leg strength (*r* = 0.36, *p* = 0.04). 

A moderate positive association was found between reactive strength and the deep squat (*r* = 0.57, *p* < 0.01; [20]). Lloyd et al. [20] found a moderate positive association between reactive strength and the hurdle step (*r* = 0.46, *p* < 0.01). Lloyd et al. [20] also found a large positive association between reactive strength and the in-line lunge (*r* = 0.70, *p* < 0.01) and they also found that the in-line lunge predicted reactive strength (adjusted R^2^ = 0.68). Only Lloyd et al. [20] found moderate positive associations between reactive strength and active straight leg raise (*r* = 0.65, *p* < 0.01) and rotary stability (*r* = 0.58, *p* < 0.01) while finding that the trunk stability push-up predicted reactive strength (adjusted R^2^ = 0.68).

#### 3.5.5. Balance

Four studies investigated balance/stability-based performance outcomes (*n =* 12) and total FMS© scores. Kramer et al. [27] found moderate positive associations between total FMS© and the lower quarter y-balance test left (LQYBTL; *r* = 0.45, *p* < 0.01) and lower quarter y-balance test total (LQYBTT; *r* = 0.42, *p* < 0.052) for males, and LQYBTL (*r* = 0.45, *p* < 0.05) and LQYBTT (*r* = 0.41, *p* < 0.05) for females. Contrastingly, Smith et al. [50] found no association between total FMS© and LQYBTL (*r* = −0.139, *p* = 0.42) or LQYBTR (*r* = −0.085, *p* = 0.623). Smith et al. [50] also found no associations between total FMS© scores and the BESS instrumental (*r* = 0.058, *p* = 0.737) and BESS clinical (*r* = 0.145, *p* = 0.398).

Only one study [45] investigated balance/stability-based outcomes and the individual movements of the FMS© Chang et al. [45] found a moderate positive association between the anterior reach maximum and the deep squat (*r* = 0.47, *p* < 0.05), a moderate positive association between the posterior medial reach maximum and the hurdle step (*r* = 0.52, *p* < 0.05), and a moderate positive association between the posterior lateral reach maximum and the hurdle step (*r* = 0.42, *p* < 0.05). Chang et al. [45] also found a moderate positive association between the anterior reach maximum and the in-line lunge (*r* = 0.53, *p* < 0.05), and a weak negative association between the posterior lateral reach maximum and rotary stability (*r* = −0.23, *p* < 0.05).

#### 3.5.6. Endurance

Five endurance-based outcomes were sourced from the included studies. Only two studies investigated the relationship between the total FMS© score and an endurance-based performance outcome [40,47]. Bennett et al. [40] found a weak positive association between total FMS© and predicted VO_2_Max (The maximum volume of oxygen consumption the body can use during exercise) (*r* = 0.22, *p* < 0.05). Yildiz [47] found a strong positive association between the crunch and total FMS^©^ (*r* = 0.75, *p* < 0.001).

When looking at the individual FMS© movements, Pfeifer [49] found that females with higher VO_2_Max (F = 3.40, *p* = 0.042) and muscular endurance (F = 4.69, *p* = 0.013) had a better hurdle step. Bennett et al. [40] found a weak negative association between predicted VO_2_Max and hurdle step (*r* = −0.02, *p* < 0.05). Pfeifer [47] found that males who had a better VO_2_Max also had a better in-line lunge (F = 4.80, *p* = 0.015) and Bennett et al. [40] found a weak positive association between predicted VO_2_Max and the in-line lunge (*r* = 0.133, *p* < 0.05).

#### 3.5.7. Flexibility

Two flexibility-based outcomes were found in the included studies. Only one study investigated the relationship between total FMS© and a flexibility-based performance outcome [47]. Yildiz [47] found a strong positive association between sit and reach and total FMS© (*r* = 0.74, *p* ≤ 0.001). No study investigated a flexibility-based performance outcome with any individual FMS^©^ movement.

#### 3.5.8. Sport-Specific Skills

No study investigated the relationship between sport-specific skills and total FMS© scores. However, Krkeljas & Kovac [44] investigated five karate movements and the individual movements of the FMS©. The authors found that the triangle step and the Gedan barai/jaku zuki had a moderate positive association with the deep squat (*r* = 0.43, *p* < 0.05, *r* = 0.40, *p* < 0.05, respectively). The Gedan barai/jaku zuki had a moderate negative association with the hurdle step (*r* = −0.61, *p* < 0.05) while the gedan barai had a moderate positive association with the hurdle step (*r* = 58, *p* < 0.05). The mawashi geri had a moderate positive association with the active straight leg raise (*r* = 0.54, *p* < 0.05). Three skills had moderate negative associations with the trunk stability push-up: the side-step (*r* = −0.50, *p* < 0.5), the triangle step (*r* = −0.56, *p* < 0.05), and the gedan barai/jaku zuki (*r* = −0.59, *p* < 0.05).

## 4. Discussion

This systematic review draws together the extant literature relating to the association between FMS© and athletic performance in youth. Given the widespread use of the FMS© as a tool to assess movement competence in both sports and health settings [19,22,52] and the apparent conflation between scores on the FMS© and measures of athletic performance, this review represents an important contribution to the literature. This review provides clarity for sports scientists, sports therapists, coaches, and public health professionals on the extent to which scores on the FMS© actually relate to athletic performance in youth. Such information is of critical importance in terms of LTAD models and professionals working on improving or assessing movement competency and understanding how FMS© may or may not be related to different aspects of athletic performance. In fact, it is important to be clear that some FMS© movements may not correlate with particular athletic performance tests. This is because there is a poor internal consistency and low correlation between the seven FMS© movements; they do not assess the same underlying variable, which has been reported by others [2,3,53,54]. As a result, we should not expect that all FMS© movements will associate with the athletic performance outcomes we have included here. 

Broadly, the results of this systematic review suggest that children and youth who score highly on the FMS© also tend to have better scores for agility, running speed, strength, and cardiovascular endurance. Without more definitive evidence, it would be imprudent to speculate on the reasons behind why there were associations between FMS© and agility, running speed, strength, and cardiovascular endurance but no associations with power and flexibility. To date, there has been no attempt to synthesise evidence relating to FMS© scores and athletic performance tasks. This paper has sought to examine this and provides a starting point that moves our understanding forward. However, further exploration is required as there is still a lot we do not know about the association between FMS© and athletic performance. However, the strength of association between FMS© and agility, running speed, strength, and cardiovascular endurance varies, as evidenced in Appendix B and Appendix C. There is some evidence that those who score highly on the FMS© also have better performance scores on tests of balance. However, the majority of associations reported by studies within this systematic review were weak to moderate in nature. The notion that scores on the FMS© might be associated with performance on various tests of athletic performance is not surprising, nor is our finding that some tests of athletic performance are associated with FMS^©^ scores. Theoretically, functional movement underpins performance in athletic tasks [17]. Using Cook’s performance pyramid conceptual model, having ‘good’ functional movement would enable a more optimum development of functional performance, which is represented via scores in athletic performance tasks, such as jump height or distance, running speed, flexibility and so forth. We would therefore hypothesise that scores on the FMS© should relate to scores on various tests of athletic performance. It is however important to note that FMS© scores are not a test of athletic performance. In part, they are not the same due to the fact that the FMS© movements have poor internal consistency and do not assess the same underlying variables, as mentioned earlier [2,3,53,54]. It is therefore important for coaches and practitioners to remember that the seven tests which comprise the total FMS© score are different from each other and while the FMS© assesses functional movement, it does not assess functional performance.

There is widespread consensus that lower scores on the FMS© reflect poorer functional movement [11] and the results of the current systematic review would support this assertion by proxy via the relationship between FMS© and athletic performance tests that are included in this systematic review. However, the magnitude of the relationship between FMS© and the different athletic performance tests in the current review, being only weak to moderate, would suggest that there are other factors aside from scores on the FMS© that might influence athletic performance. One reason for these small to moderate associations could be attributed to the scoring systems. The FMS© places performers into four categories (0–3) while all measures of athletic performance were measured on continuous scales. Although no ceiling effects were reported in any of the included studies within this review, the broad categorisation of the FMS© may limit the associations that might be drawn in relation to athletic performance. Regardless, scores on the FMS^©^ should not be taken as a proxy for athletic performance, rather coaches, exercise scientists, and athletes need to be aware that the FMS© assesses functional movement, and while functional movement relates to athletic performance, the two constructs are not the same.

It is important to note that there was a considerable variation in the different athletic performance measures used in the studies included in the current systematic review. This makes it difficult to make strong conclusions in relation to how FMS© scores are related to athletic performance. For example, different studies assessed different sprint distances in relation to FMS© scores, whilst others examined repeated sprint ability. While both considered measures of ‘speed’, repeat sprint ability taps into a slightly different aspect of movement than an assessment of straight-line speed over one trial. Similar issues were evident where strength was assessed but, in some studies, isometric strength (handgrip) was examined, and in others, more dynamic measures of strength (concentric strength) were employed, and in others again, measures of muscular endurance (sit-ups/press-ups) were employed but all labelled as ‘strength’ when such different measures tap into different aspects of muscular system performance. Likewise, while the importance and effect of biological maturation on athletic performance during youth is well established [55], it was surprising that maturation was seemingly overlooked as an important variable in those studies that examined associations between FMS© scores and athletic performance measures in children and youth. Only one study [20] considered maturation in their analysis. Furthermore, given the noted variability in the association between FMS^©^ and BMI, potentially due to maturation, as noted in the recent review by O’Brien et al. [25], it is a key consideration for future research in this area with youth participants, that biological maturation is captured and considered in any analysis of the association between functional movement and athletic performance. However, while the recent review by O’Brien et al. [25] specifically examined sex and age differences in FMS© scores in youth, the current study is qualitatively different in focus, examining any associations between FMS© scores and measures of athletic performance in adolescents. 

In relation to the quality assessment of studies included in this review, only one of the studies [45] included a justification for their sample size, with the majority of participants in each study selected using convenience sampling. Likewise, no study attempted to account for non-responders and, consequently, questions around bias due to response rate cannot be determined. Only four of the included studies reported data demonstrating internal consistency of their FMS© and athletic performance measures with a further two studies reporting internal consistency data for the FMS© only. In the future, further research could enhance the robustness of their work by ensuring studies are adequately powered, that attempts are made to describe non-responders, and that the internal consistency/reliability of all measures are reported.

There are some limitations of the present study. We examined the association between FMS© scores and performance outcomes, as described by the LTAD or YPD models. It is possible other constructs that relate to athletic performance (e.g., cognition) might relate to functional movement. However, we sought to anchor search terms with those models of athletic performance that have an established theoretical base. In addition, there are some examples of intervention studies that have used various functional movement-based interventions with youth. The extant literature on this topic was overwhelmingly cross-sectional in nature and, as a consequence, the current systematic review is limited to examining the association of FMS© with athletic performance. While this provides a robust foundation to understand how FMS© scores relate to athletic performance in youth, there is a need to examine different types of intervention and how these might impact athletic performance through the development of FMS© and for different stages of development (e.g., children vs youth, pre vs post PHV). Prior work, including systematic reviews, have also suggested a composite score of below 14 as a cut-off for increased injury risk [11,56,57]. This cut-off, and its widespread use, has been considered controversial and has been criticised as an arbitrary cut-off value of 14 for representing greater injury risk, and is not supported in the literature [52,58], hence why the current review did not examine this aspect of the FMS© within the current review. Moreover, the current review examined studies that used the FMS© with a 0–21 scoring scale. Although a 100-point FMS© scale, developed [18], is available and suggested as the more sensitive metric for use in research, no studies examined the association between FMS scores and athletic performance using the 100-point scale. The 100-point scoring scheme scores individual criteria for each of the seven FMS© tests giving a score that reportedly allows greater sensitivity in detecting movement deficits and improves intervention specificity [18,59]. As it stands, it remains unknown whether using the 100-point FMS scale strengthens the association between FMS© scores and scores on tests of athletic performance.

As this paper is one of the first to synthesise evidence relating to FMS© and performance outcomes, future work examining how scores on the FMS© might underpin athletic performance during youth would be useful in unpacking the relationship between the FMS© and subsequent performance outcomes. Such work should also seek to account for the maturation status of performers. The distinct lack of studies controlling or accounting for maturation when examining the relationship between FMS© scores and athletic performance tests in youth prevents an in-depth understanding of how FMS© relates to performance in this population. Further work should be conducted to investigate the relationship between flexibility and the individual movements of the FMS© as this review highlighted a distinct lack of investigation in this area.

## 5. Conclusions

This systematic review suggests that children and youth who score higher on the FMS© are also more likely to score highly in tests related to agility, running speed, strength, and cardiovascular endurance and some, albeit sparser, evidence that this is the case for tests of balance also. The strength of the association varies but are broadly weak to moderate in magnitude. From its inception, the proponents of the FMS© describe it as a series of “seven tests that utilise a variety of basic positions and movements, which are thought to provide the foundation for more complex athletic movements to be performed efficiently” ([60], p. 148) and while functional movement and functional performance are conceptually related, the FMS© should not be considered as a test of performance or to predict performance. The magnitude of relationships identified in the present review would support this assertion. This point appears to have been lost in the expanding volume of literature that has examined functional movement alongside athletic performance. The present review provides an opportunity to reign in this notion and be clear on what the FMS© is and is not. These results provide evidence that further longitudinal inquiry is needed into the utility of FMS© and a measure of athletic performance while considering maturational status. It is important for sports scientists, sports therapists, coaches, and public health professionals to recognise that the FMS© does not assess athletic performance and while there is a relationship between FMS© scores and tests of athletic performance, the two should be considered conceptually different constructs.

## Figures and Tables

**Table 1:** Outcome categories by functional movement and athletic performance.

Functional Movement (from the FMS©)	Outcomes
Total	Composite scores
Domain-specific	FMSmove
	FMSflex
	FMSstab
Individual FMS movements	deep squat
	hurdle step
	in-line lunge
	shoulder mobility
	active straight leg-raise
	trunk stability
	rotary stability
Athletic performance	
	jump height or distance
	speed
	strength
	balance
	agility
	power
	fitness
	physical performance
	athletic performance
	muscular endurance
	cardiovascular endurance

Note. FMS = Functional Movement Screen, flex = flexibility, stab = stability.

**Table 2:** Demographic data of included studies.

Study	*n*	Sex (*n*)	Overall Age Mean ± SD (*n*)	Min.–Max. Age	Status/Sport	Study Design
Lloyd et al., 2015 [20]	30	M	“Under 11”: 11.20 ± 0.50 (10) “Under 13”: 13.20 ± 0.20 (9) “Under 15”: 15.60 ± 0.70 (11)	NR	Professional football club/Soccer	Cross-sectional
Kramer et al., 2019 [27]	56	M (28) F (28)	16.40 ± 0.10	NR	High school students/Sport NR	Cross-sectional
Bennett et al., 2021 [40]	981	M	17.40 ± 3.40	15–18	Elite Under 18 competitors/Australian Football	Cross-sectional and longitudinal
Campa et al., 2019 [41]	36	M	16.60 ± 50	NR	Elite and Sub-Elite/Soccer	Observational
Silva et al., 2017 [42]	48	NR	“Under 16”: 15.78 ± 0.52 (22) “Under 19”: 17.32 ± 0.48 (26)	NR	National competitive players/Soccer	Cross-sectional
Bakalľár et al., 2020 [43]	41	M	“Under 12”: 11 (15) ^a^ “Under 14”: 13 (14) ^a^ “Under 16”: 15 (12) ^a^	11–15	Local soccer academy/Soccer	Cross-sectional
Krkeljas et al., 2021 [44]	20	NR	12.20 ± 1.90	10–15	Belt colour orange to brown/Karate (7.50 ± 4.42 years’ experience)	Cross-sectional
Chang et al., 2020 [45]	32	NR	16.06 ± 0.21	NR	Junior athletes (school sports teams)/Volleyball (*n =* 11) Basketball (*n =* 12) Handball (*n =* 9)	Observational
GÜNay et al., 2017 [46]	93	M (50) F (43)	NR	NR	Olympic Swimmer Development Camp (National Federation of Swimming)/Swimming	Cross-sectional
Yildiz et al., 2018 [47]	32	M (20) F (12)	16.06 ± 0.90	15–18	Blue belt or higher/Karate	Cross-sectional
Bond et al., 2015 [48]	50	M (21) F (29)	NR	11–16	National Amateur Swimming Association beacon squad/Swimming	Cross-sectional
Pfeifer et al., 2017 [49]	136	M (63) F (73)	16.01 ± 1.35	11–18	Public/private high schools and local sports organisations/Football (*n =* 40), Soccer (M = 23, F = 39), Volleyball (F = 18), Lacrosse (F = 10), ‘Other’ (F = 6)	Cross-sectional
Smith et al., 2017 [50]	94	M	15.50 ± 1.17	13–18	High school athletes/Football (53), Baseball (1), Soccer (40)	Cross-sectional

Note. ^a^ = no mean reported, M = male, F = female, NR *=* Not reported.

**Table 3:** Risk of bias summary.

	Studies (Yes/No/Don’t Know/N/A)												
	20	27	40	41	42	43	44	45	46	47	48	49	50
**Introduction**													
Were the aims/objectives of the study clear	Y	Y	Y	Y	Y	Y	Y	Y	Y	Y	Y	Y	Y
**Methods**													
Was the study design appropriate for the stated aim(s)?	Y	Y	Y	Y	Y	Y	Y	Y	Y	Y	Y	Y	Y
Was the sample size justified?	N	N	N	N	N	N	N	Y	N	N	N	N	N
Was the target/reference population clearly defined? (Is it clear who the research was about?)	Y	Y	Y	Y	N	N	N	N	Y	N	Y	Y	Y
Was the sample frame taken from an appropriate population base so that it closely represented the target/reference population under investigation?	Y	Y	Y	Y	Y	DK	Y	Y	Y	DK	Y	Y	Y
Was the selection process likely to select subjects/participants that were representative of the target/reference population under investigation?	Y	Y	Y	Y	Y	DK	Y	Y	Y	DK	Y	Y	Y
Were measures undertaken to address and categorise non-responders?	N	N	N	N	N	N	N	N	N	N	N	N	N
Were the risk factor and outcome variables measured appropriate to the aims of the study?	Y	Y	Y	Y	Y	Y	Y	Y	Y	Y	Y	Y	Y
Were the risk factor and outcome variables measured correctly using instruments/measurements that had been trialled, piloted or published previously?	Y	Y	Y	Y	Y	Y	Y	Y	Y	DK	Y	Y	Y
Is it clear what was used to determine statistical significance and/or precision estimates? (e.g., *p* values, CIs)	Y	Y	Y	Y	Y	Y	Y	Y	Y	N	Y	Y	Y
Were the methods (including statistical methods) sufficiently described to enable them to be repeated?	Y	N	Y	Y	Y	Y	N	Y	N	N	N	Y	Y
**Results**													
Were the basic data adequately described?	Y	Y	Y	Y	Y	Y	Y	N	Y	N	Y	Y	Y
Does the response rate raise concerns about non-response bias?	DK	DK	DK	DK	DK	DK	DK	DK	DK	DK	DK	DK	DK
If appropriate, was information about non-responders described?	N	N	N	N	N	N	N	N	N	N	N	N	N
Were the results internally consistent?	DK	N	DK	DK	DK	Y	DK	Y	N	DK	Y	Y	N
Were the results for the analyses described in the methods, presented?	Y	Y	Y	Y	Y	Y	Y	Y	Y	N	Y	Y	Y
**Discussion**													
Were the authors’ discussions and conclusions justified by the results?	Y	Y	Y	Y	Y	Y	N	Y	Y	Y	Y	Y	Y
Were the limitations of the study discussed?	Y	N	Y	N	Y	Y	N	Y	N	N	Y	Y	Y
**Other**													
Were there any funding sources or conflicts of interest that may affect the authors’ interpretation of the results?	N	DK	DK	N	N	N	DK	N	DK	DK	N	DK	N
Was ethical approval or consent of participants attained?	Y	Y	Y	Y	Y	Y	Y	Y	Y	Y	Y	Y	Y

Note. DK = Don’t know, N/A = Not Applicable.

## Appendix A

**Table A1:** Means, standard deviations, and range for FMS^©^ outcome.

Grouped FMS©		Individual FMS©												
		Outcome by Age				Outcome by Gender			Overall Outcome					
	Campa et al., 2019 [41]		Bakalľár et al., 2020 [43]	Lloyd et al., 2015 [20]	Silva et al., 2017 [42]	GÜNay et al., 2017 [46]	Kramer et al., 2019 [27]	Pfeifer et al., 2017 [49]	Bond et al., 2015 [48]	Bennett et al., 2021 [40]	Chang et al., 2020 [45]	Krkeljas et al., 2021 [44]	Smith et al., 2017 [50]	Yildiz et al., 2018 [47]
Total FMS^©^ scores	Elite = 13.50 (2.10) Sub-elite = 12.40 (1.60)	Total	11 = 15.70 ^a^ (1.90) 13 = 16.00 ^a^ (1.60) 15 = 17.20 ^a^ (1.20)	U11 = 12.00 (1.50) U13 = 12.50 (3.00) U16 = 16.00 (2.00)	U16 = 13.87 (2.93) U19 = 14.96 (2.07)	M = 16.13 (2.32) F = 17.71 (1.65)	M = 12.70 (2.60) F = 14.20 (2.20)	M = 12.62 (3.06) [6–18] F = 14.10 (1.88) [9–18]	15.90 (1.40) M = 15.20 (0.26) F = 16.30 (0.22)	13.78 (2.53)	12.18 (2.02)	13.50 (8.10)	16.00 ^a^ [9–21]	15.86 (2.06)
FMS_move_	Elite = 5.90 (0.80) Sub-elite = 4.80 (0.90)	DS	11 = 2 13 = 2 15 = 2		U16 = 2.13 (0.69) U19 = 2.08 (0.40)	M = 2.47 (0.73) F = 2.59 (0.49)		M = 1.70 (0.56) [1–3] F = 1.80 (0.67) [1–3]		1.65 (0.72)	1.68 (0.78)	1.96		
		HS	11 = 2 13 = 2 15 = 2		Right leg U16 = 1.70 (0.47) U19 = 1.84 (0.47) Left leg U16 = 1.57 (0.51) U19 = 1.84 (0.47)	M = 1.93 (0.62) F = 2.12 (0.63)		M = 1.65 (0.54) [0–3] F = 1.91 (0.42) [1–3]		1.90 (0.52)	2.32 (0.48)	2.12		
		ILL	11 = 2 13 = 2 15 = 2		Right leg U16 = 2.00 (0.80) U19 = 2.52 (0.71) Left leg U16 = 2.04 (0.93) U19 = 2.20 (0.87)	M = 1.93 (0.76) F = 2.12 (0.78)		M = 2.13 (0.66) [1–3] F = 2.32 (0.53) [1–3]		2.04 (0.58)	1.45 (0.74)	1.84		
FMS_flex_	Elite = 4.30 (0.90) Sub-elite = 3.70 (1.40)	SM	11 = 3 13 = 3 15 = 3		Right U16 = 2.17 (0.89) U19 = 2.52 (0.71) Left U16 = 2.04 (0.93) U19 = 2.20 (0.87)	M = 2.36 (0.78) F = 2.61 (0.53)		M = 2.02 (1.00) [0–3] F = 2.68 (0.62) [1–3]		2.42 (0.91)	1.64 (1.00)	2.88		
		ASLR	11 = 3 13 = 2 15 = 2.50		Right U16 = 2.57 (0.66) U19 = 2.60 (0.50) Left U16 = 2.48 (0.59) U19 = 2.60 (0.50)	M = 2.43 (0.97) F = 2.93 (0.24)		M = 1.87 (0.61) [1–3] F = 2.32 (0.75) [1–3]		2.08 (0.77)	1.86 (0.35)	1.84		
FMS_stab_	Elite = 4.10 (0.10) Sub-elite = 3.80 (0.40)	TSPU	11 = 2 13 = 3 15 = 3		U16 = 2.04 (0.82) U19 = 2.20 (0.91)	M = 2.86 (0.35) F = 2.87 (0.66)		M = 1.60 (0.77) [0–3] F = 1.46 (0.77) [1–3]		1.98 (1.01)	2.32 (1.09)	1.92		
		RS	11 = 2 13 = 2 15 = 2		Right U16 = 1.91 (0.42) U19 = 1.92 (0.40) Left U16 = 1.83 (0.49) U19 = 2.00 (0.00)	M = 2.13 (0.67) F = 2.42 (0.57)		M = 1.65 (0.60) [0–3] F = 1.91 (0.38) [1–3]		1.86 (0.47)	1.50 (0.80)	2.00		

Note. Standard deviations in brackets, range in square brackets, ^a^ = median, M = Male, F = Female, DS = Deep squat, HS = Hurdle step, ILL = In-line lunge, SM = Shoulder mobility, ASLR *=* Active straight-leg raise, TSPU = Trunk stability push-up, RS = Rotary stability.

## Appendix B

**Table A2:** Correlational relationships between total FMS^©^ scores and athletic performance outcomes.

	Lloyd et al., 2015 [20]	Kramer et al., 2019 [27]	Bennett et al., 2021 [40]	Campa et al., 2019 [41]	GÜNay et al., 2017 [46]	Yildiz et al., 2018 [47]	Bond et al., 2015 [48]	Smith et al., 2017 [50]
Total FMS©	Squat jump (*r* = 0.66, *p* < 0.01) Reactive strength (*r* = 0.74, *p* < 0.01) Reactive agility (*r* = −0.54, *p* < 0.01)	M LQYBTL (*r* = 0.45, *p* < 0.01) LQYBTT (*r* = 0.42, *p* < 0.05) Agility (*r* = −0.47, *p* < 0.01) F LQYBTL (*r* = 0.45, *p* < 0.05) LQYBTT (*r* = 0.41, *p* < 0.05)	5 m sprint (*r* = −0.13, *p* < 0.05) Agility (*r* = −0.13, *p* < 0.05) PredV02max (*r* = 0.22, *p* < 0.05)	RSAM (*r* = −0.68, *p* < 0.001) RSAB (*r* = −0.58, *p* < 0.001)	M swim 200 m time (*r* = 0.014, *p* = 0.92) F swim 200 m time (*r* = 0.022, *p* = 0.89)	Squat jump (*r* = 0.52, *p* = 0.002) Sit and reach (*r* = 0.74, *p* = 0.000) Crunch (*r* = 0.75, *p* = 0.000) Back and leg strength (*r* = 0.36, *p* = 0.04) LP-R (*r* = 0.55, *p* = 0.001) LP-L (*r* = 0.57, *p* = 0.001) Core stability (*r* = 0.75, *p* = 0.000)	100 m freestyle swim (*r* = −0.333, *p* < 0.05) Sig. main effect for swim speed (fast v slow) (F = 8.20, *p* = 0.005)	BESS instrumental (*r* = 0.058, *p* = 0.737) BESS clinical (*r* = 0.145, *p* = 0.398) Y-Balance right (*r* = −0.085, *p* = 0.623) Y-Balance left (*r* = −0.139, *p* = 0.42)
FMS_move_				RSAM (*r* = −0.36, *p* < 0.05) RSAB (*r* = −0.55, *p* < 0.001)				

Note. BESS = Balance error scoring system, LP-L = Lateral plank-left, LP-R *=* Lateral plank-right, LQYBTL = Lower quarter Y-balance test left, LQYBTT = Lower quarter Y-balance test total, RSAM = Repeated sprint ability mean time, RSAB = Repeated sprint ability best time.

## Appendix C

**Table A3:** Correlational and regression relationships between individual FMS^©^ movements and athletic outcomes, and ANOVA results.

	Lloyd et al., 2015 [20]	Kramer et al., 2019 [27]	Bennett et al., 2021 [40]	Campa et al., 2019 [41]	GÜNay et al., 2017 [46]	Yildiz et al., 2018 [47]	Bond et al., 2015 [48]	Smith et al., 2017 [50]
Total FMS©	Squat jump (*r* = 0.66, *p* < 0.01) Reactive strength (*r* = 0.74, *p* < 0.01) Reactive agility (*r* = −0.54, *p* < 0.01)	M LQYBTL (*r* = 0.45, *p* < 0.01) LQYBTT (*r* = 0.42, *p* < 0.05) Agility (*r* = −0.47, *p* < 0.01) F LQYBTL (*r* = 0.45, *p* < 0.05) LQYBTT (*r* = 0.41, *p* < 0.05)	5 m sprint (*r* = −0.13, *p* < 0.05) Agility (*r* = −0.13, *p* < 0.05) PredV02max (*r* = 0.22, *p* < 0.05)	RSAM (*r* = −0.68, *p* < 0.001) RSAB (*r* = −0.58, *p* < 0.001)	M swim 200 m time (*r* = 0.014, *p* = 0.92) F swim 200 m time (*r* = 0.022, *p* = 0.89)	Squat jump (*r* = 0.52, *p* = 0.002) Sit and reach (*r* = 0.74, *p* = 0.000) Crunch (*r* = 0.75, *p* = 0.000) Back and leg strength (*r* = 0.36, *p* = 0.04) LP-R (*r* = 0.55, *p* = 0.001) LP-L (*r* = 0.57, *p* = 0.001) Core stability (*r* = 0.75, *p* = 0.000)	100 m freestyle swim (*r* = −0.333, *p* < 0.05) Sig. main effect for swim speed (fast v slow) (F = 8.20, *p* = 0.005)	BESS instrumental (*r* =.058, *p* = 0.737) BESS clinical (*r* = 0.145, *p* = 0.398) Y-Balance right (*r* = −0.085, *p* = 0.623) Y-Balance left (*r* = −0.139, *p* = 0.42)
FMS_move_				RSAM (*r* = −0.36, *p* < 0.05) RSAB (*r* = −0.55, *p* < 0.001)				

Note. BESS = Balance error scoring system, LP-L = Lateral plank-left, LP-R *=* Lateral plank-right, LQYBTL = Lower quarter Y-balance test left, LQYBTT = Lower quarter Y-balance test total, RSAM = Repeated sprint ability mean time, RSAB = Repeated sprint ability best time.
